# *Neurospora* Heterokaryons with Complementary Duplications and Deficiencies in Their Constituent Nuclei Provide an Approach to Identify Nucleus-Limited Genes

**DOI:** 10.1534/g3.115.017616

**Published:** 2015-04-20

**Authors:** Dev Ashish Giri, S. Rekha, Durgadas P. Kasbekar

**Affiliations:** * Centre for DNA Fingerprinting and Diagnostics, Hyderabad 500001, India; † Graduate Studies, Manipal University, Manipal 576104, Karnataka, India

**Keywords:** chromosome translocation, introgression, pseudohomothallism, RIP, MSUD

## Abstract

Introgression is the transfer of genes or genomic regions from one species into another via hybridization and back-crosses. We have introgressed four translocations (*EB4*, *IBj5*, *UK14-1*, and *B362i*) from *Neurospora crassa* into *N. tetrasperma*. This enabled us to construct two general types of heterokaryons with *mat-A* and *mat-a* nuclei of different genotypes: one type is [*T* + *N*] (with one translocation nucleus and one normal sequence nucleus), and the other is [*Dp* + *Df*] (with one nucleus carrying a duplication of the translocation region and the other being deleted for the translocation region). Self-crossing these heterokaryons again produced [*T* + *N*] and [*Dp* + *Df*] progeny. From conidia (vegetative spores) produced by the heterokaryotic mycelia, we obtained self-fertile (heterokaryotic) and self-sterile (homokaryotic) derivative strains. [*T* + *N*] heterokaryons produced homokaryotic conidial derivatives of both mating types, but [*Dp* + *Df*] heterokaryons produced viable conidial homokaryons of only the mating type of the *Dp* nucleus. All four [*T* + *N*] heterokaryons and three [*Dp* + *Df*] heterokaryons produced both self-sterile and self-fertile conidial derivatives, but the [*Dp(B362i)* + *Df(B362i)*] heterokaryons produced only self-sterile ones. Conceivably, the *Df(B362i)* nuclei may be deleted for a nucleus-limited gene required for efficient mitosis or nuclear division, and whose deficit is not complemented by the neighboring *Dp(B362i)* nuclei. A cross involving *Dp(EB4)* showed repeat-induced point mutation (RIP). Because RIP can occur in self-crosses of [*Dp* + *Df*] but not [*T* + *N*] heterokaryons, RIP alteration of a translocated segment would depend on the relative numbers of [*Dp* + *Df*] *vs.* [*T* + *N*] ancestors.

Perkins (1997) described three elementary chromosome translocation (*T*) types in *Neurospora crassa*, namely, insertional (*IT*), quasiterminal (*QT*), and reciprocal (*RT*). *IT*s transfer a segment of a donor chromosome into a recipient chromosome without any reciprocal exchange (Figure 1); *QT*s transfer a distal segment of a donor chromosome to the tip of a recipient chromosome, distal to any essential gene, and presumably the donor chromosome breakpoint is capped with the tip from the recipient chromosome; and *RT*s reciprocally interchange the terminal segments of two chromosomes. Other chromosome rearrangements are essentially variants of these, *e.g.*, an intrachromosomal transposition (*Tp*) is an *IT* in which the same chromosome is both donor and recipient, an inversion (*In*) is a *Tp* in which a chromosome segment is re-inserted in opposite orientation into the site from which it was derived, and there are complex rearrangements such as linked *RT* and *IT*. Three breakpoint junctions define an *IT*: junction A created by the deletion on the donor chromosome, and junctions B and C (proximal and distal), created by the insertion into the recipient chromosome; however, two breakpoint junctions define a *QT* or *RT*: junction A, between the breakpoint-proximal segment on the donor chromosome and the tip from the recipient chromosome, and junction B, between the breakpoint-proximal sequence on the recipient chromosome and the donor segment grafted onto it (Singh *et al.* 2010). In the cross of a translocation strain with normal sequence, the chromosomes can segregate in one of two ways in meiosis I: alternate or adjacent 1 (see Figure 1 for *IT* × *N*). Alternate segregation produces eight parental-type ascospores (*i.e.*, 4 *IT* + 4 *N*), whereas adjacent 1 segregation produces eight nonparental ascospores, namely, four viable ascospores containing a duplication (*Dp*) of the translocation segment and four inviable ones with the complementary deficiency (*Df*) (Figure 1). Viable ascospores blacken (B), whereas inviable ones remain white (W). Therefore, alternate and adjacent 1 segregation produce, respectively, 8B:0W and 4B:4W asci (Perkins 1997). Because both segregations are equally likely, *IT* × *N* and *QT* × *N* crosses are characterized by 8B:0W = 4B:4W, whereas isosequential crosses (*i.e.*, *N* × *N* or *T* × *T*) produce mostly 8B:0W asci (Perkins 1997). In an *RT*, the two chromosomes that underwent reciprocal interchange of their terminal segments can be designated as *T^1^* and *T^2^* and their normal sequence homologs can be designated as *N^1^* and *N^2^*. In an *RT* × *N* cross, alternate segregation moves *T^1^* and *T^2^* to one spindle pole and *N^1^* and *N^2^* to the other to produce eight parental-type ascospores (*i.e.*, 4 *RT* + 4 *N*) that are viable and black. Adjacent 1 segregation moves *N^1^* and *T^2^* to one pole and *N^2^* and *T^1^* to the other to generate only inviable white ascospores bearing complementary duplications and deficiencies (*i.e.*, *Dp2*/*Df1* and *Dp1*/*Df2*), and the asci are 0B:8W. In other words, obtaining 8B:0W = 0B:8W signals an *RT* × *N*.

**Figure 1:**
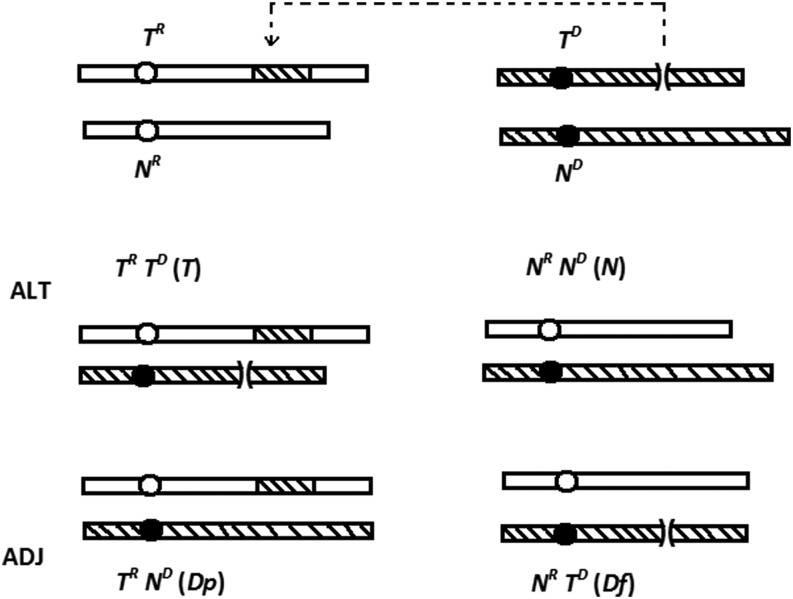
Alternate and adjacent 1 segregation in *IT* × *N*. The donor and recipient chromosomes of the *IT* are designated as *T^D^* and *T^R^* and their normal sequence homologs are designated as *N^D^* and *N^R^*. In alternate segregation (ALT), *T^D^* and *T^R^* segregate to one spindle pole, and *N^D^* and *N^R^* segregate to the other. Subsequently, meiosis II and postmeiotic mitosis generate eight parental-type nuclei, namely, four *IT* + four *N*. In adjacent 1 segregation (ADJ), *N^D^* and *T^R^* segregate to one pole and *T^D^* and *N^R^* segregate to the other to ultimately produce eight nonparental nuclei: four *Dp* + four *Df*.

*Dp* strains (*i.e.*, the viable segregants from 4B:4W asci) are recognizable by the characteristic barren phenotype of *Dp* × *N* crosses, wherein normal-looking perithecia are made, but only a few exceptional ascospores are produced (Perkins 1997). Barrenness is caused by meiotic silencing by unpaired DNA (MSUD), an RNAi-mediated process that eliminates the transcripts of any gene not properly paired with a homolog during meiosis (Shiu *et al.* 2001). Presumably, *Dp*-borne genes, including those underlying ascus and ascospore development, fail to be properly paired in a *Dp* × *N* cross, and their silencing by MSUD renders the cross barren. The breakpoint junctions of several *IT*s, *QT*s, and *RT*s were defined in our laboratory (Singh 2010; Singh *et al.* 2010). PCR with breakpoint junction-specific primers can now be used to distinguish the *Dp* progeny from their *T* and *N* siblings. *IT* progeny contain all three breakpoints (A, B, and C), *Dp* progeny contain B and C, but not A, and *N* progeny contain none. Although *Dp*s have been extensively studied (Perkins 1997; Kasbekar 2013), the use of *Df*s was limited to flagging the *Dp*-bearing 4B:4W asci. We now report the generation of [*Dp* + *Df*] heterokaryons with complementing duplications and deficiencies in their constituent nuclei. They were obtained by introgressing *N. crassa IT*s (and a *QT*) into *N. tetrasperma*. Introgression is the transfer of genes or genomic regions from one species into another (Rieger *et al.* 1991).

Eight ascospores form per ascus in *N. crassa*, whereas four are formed in *N. tetrasperma*. In both species the parental *mat A* and *mat a* nuclei fuse in the ascogenous cell to produce a diploid zygote nucleus that immediately undergoes meiosis and a postmeiotic mitosis to generate eight haploid nuclei (four *mat A* + four *mat a*). In *N. crassa* the nuclei are partitioned into the eight initially uninucleate ascospores (Raju 1980). In contrast, *N. tetrasperma* ascospores are initially binucleate, receiving a pair of nonsister nuclei (one *mat A* + one *mat a*) (Raju and Perkins 1994). Thus, *N. crassa* ascospores produce homokaryotic mycelia that are either *mat A* or *mat a* in mating type, whereas *N. tetrasperma* ascospores can produce heterokaryotic mycelia containing nuclei of both mating types. In *N. crassa*, a sexual cross perforce requires mycelia from two different ascospores, one *mat A* and the other *mat a*, thus making the lifecycle “heterothallic”; but a heterokaryotic *N. tetrasperma* mycelium from a single ascospore bearing nuclei of both mating types is competent to undergo a self-cross, making the lifecycle “pseudohomothallic.” However, a subset of conidia (vegetative spores) produced by a heterokaryotic *N. tetrasperma* mycelium can be homokaryotic by chance, and *N. tetrasperma* ascogenesis occasionally produces five or more (up to eight) ascospores by replacement of one or more dikaryotic ascospore by a pair of smaller homokaryotic ones (Raju 1992). The dominant *Eight-spore* (*E*) mutant increases the frequency of such replacement, although *E*-homozygous crosses are infertile (Calhoun and Howe 1968). Mycelia from homokaryotic conidia or ascospores can cross with like mycelia of the opposite mating type. Therefore, *N. tetrasperma* is actually a facultatively heterothallic species.

Although [*mat A* + *mat a*] heterokaryons can form quite easily in *N. tetrasperma*, their formation is prevented in *N. crassa* by mating type–mediated vegetative incompatibility (Newmeyer 1970). The *N. crassa* allele of the *tolerant* gene (*tol^C^*) is a key regulator of this incompatibility (Shiu and Glass 1999). If *tol^C^* is replaced either by a recessive allele *tol* or by the *N. tetrasperma* allele (*tol^T^*), then heterokaryons of genotype [(*tol mat A*) + (*tol mat a*)] can form in *N. crassa* and are stable, provided that they are homokaryotic for *het* loci that determine other vegetative heterokaryon incompatibilities (Smith and Lafontaine 2013). *N. crassa* strains of the same mating type and sharing the same alleles at the other *het* loci can fuse to form a culture that has both nuclear types in a common cytoplasm. Heterokaryon formation between two strains with different auxotrophic mutations can be “forced” by plating them together on minimal medium. The *helper-1* strain (genotype *a^m1^ ad-3B cyh-1*) is capable of forming vigorous heterokaryons with either of the mating type strains of the standard laboratory Oak Ridge (OR) background, because the *mat a* allele *a^m1^* is inactive and does not elicit mating type–mediated vegetative incompatibility. The *a^m1^* allele also makes the *helper-1* component of such a heterokaryon a passive partner when the heterokaryon is used in a cross (Perkins *et al.* 2001). We have used *helper-1* to genetically map the *fmf-1* mutation (Iyer *et al.* 2009), which has a unique female-sterile and male-sterile phenotype. Heterokaryons have also been used for the analysis of complementation, and to “rescue” recessive lethal mutations. Additionally, heterokaryosis in *N. crassa* (Davis 1960) and *Penicillium cyclopium* (Jinks 1952) has long been known to confer phenotypic plasticity that enables the fungus to respond to changes in environmental conditions by changes in the ratios of the constituent nuclei, but how this happens is still not understood. Recent studies from Dr. Hanna Johannesson’s laboratory have shown that the *mat A*/*mat a* nuclear ratio can change during the *N. tetrasperma* lifecycle (Samils *et al.* 2014). The ratio is biased for *mat A*-nuclei during mycelial growth and becomes more balanced only during sexual development. There was also a bias in expression for *mat A*–linked genes during mycelial growth that switched during the sexual stage into a bias for genes of the *mat a* nuclei. These findings were interpreted to suggest that *N. tetrasperma mat A* and *mat a* nuclei have co-evolved to optimize their relative fitness in the heterokaryon by altering their ratios and by regulating gene expression. Previous studies from this group showed that that wild-isolated *N. tetrasperma* strains from United Kingdom generally produced a greater proportion of homokaryotic conidia than strains isolated from New Zealand, and some even showed a strong bias in the homokaryotic conidia for one of the mating types (*e.g.*, 0 *mat A*: 17 *mat a*; 7 *mat A*: 0 *mat a*; and 0 *mat A*: 9 *mat a*). However, all the strains made self-fertile conidia (Corcoran *et al.* 2012). The high proportion of single-mating-type conidia was attributed to a putative nonrandom distribution of nuclei in the heterokaryotic mycelium and/or stronger ability of certain nuclei to be packaged into conidia.

Here, we have introgressed three *N. crassa IT*s (*EB4*, *IBj5*, and *B362i*) and one *QT* (*UK14-1*) into *N. tetrasperma*, and we have shown that *T* × *N* crosses can produce both [*T* + *N*] and [*Dp* + *Df*] heterokaryotic progeny. The [*Dp* + *Df*] progeny allowed us to ask whether the *Df* deletes any gene with a nucleus-limited function. A gene may be considered to be nucleus-limited if nuclei bearing its null allele (*Δ*) are not complemented by wild-type nuclei (*WT*) in a [*Δ* + *WT*] heterokaryon (Kasbekar 2014). If a *Df* deletes a nucleus-limited gene, then one might expect to see a phenotype difference between [*T* + *N*] and [*Dp* + *Df*] heterokaryons. Although no nucleus-limited genes have been reported as yet, their existence in fungi is not ruled out, especially given the putative nucleus-limited behavior of the *N. crassa scon^c^* mutant (Burton and Metzenberg 1972), the DNA damage checkpoint signal in *Saccharomyces cerevisiae* (Demeter *et al.* 2000), and the MatIS gene silencing process in *Aspergillus nidulans* (Czaja *et al.* 2013). We found that although [*T(B362i) A*+ *N a*] heterokaryons could produce both heterokaryotic and homokaryotic conidia, the [*Dp(B362i) a* + *Df(B362i) A*] heterokaryons produced only homokaryotic conidia, possibly because a putative “nucleus-limited” gene required for efficient packaging of nuclei into conidia is absent from the *Df(B362i)* nuclei. Second, we found evidence that suggests that the *N. tetrasperma E* mutant contains a recessive mutation affecting alternate but not adjacent 1 segregation. Third, we show that a *Dp*-heterozygous cross can exhibit RIP (repeat-induced point mutation), the sexual stage-specific process that induces G:C to A:T mutations in duplicated DNA (Selker 1990).

## Materials and Methods

### Neurospora strains and general genetic manipulations

All Neurospora strains were obtained from the Fungal Genetics Stock Center, Kansas State University, Manhatten, Kansas USA, unless otherwise indicated. *N. crassa* OR *A* (FGSC 987) and OR *a* (FGSC 988) are the standard laboratory Oak Ridge strains; the translocation strains are *T*(*VR→VII*)*EB4 A* (FGSC 3046), *T(VIL→IR) IBj5 cpc-1 A* (FGSC 4433), *T(VIR > VL) UK14-1 A* (FGSC 6958), and *T(IV→I)B362i A* (FGSC 2935) [abbreviated *T(EB4)*, *T(IBj5)*, *T(UK14-1)*, and *T(B362i)*]. *T(EB4)*, *T(IBj5)*, and *T(B362i)* are *IT*s, whereas *T(UK14-1)* is a *QT*. These translocations have been described by Perkins (1997) and Singh (2010). Perkins (1997) reported that *T(IBj5)* translocates a chromosome VIL segment to IR, linked to *al-2* and *un-18*. However, Singh *et al.* (2010) showed that the VIL segment is, in fact, inserted into a chromosome IVR sequence. Therefore, *T(IBj5)* might include an additional rearrangement (possibly an *RT*) involving IVR and IR, whose breakpoints are as yet undetermined. Because *T(B362i)* also involves chromosomes I and IV, the cross *T(IBj5)* × *T(B362i)* would resemble an *RT*-heterozygous cross.

The semi-dominant MSUD suppressor strains *Sad-1 A* (FGSC 8740) and *Sad-1 a* (FGSC 8741) were gifted by the late Robert L. Metzenberg and are described by Shiu *et al.* (2001). The *sad-1* locus encodes an RNA-dependent RNA polymerase essential for MSUD, and the *Sad-1* suppressor allele is presumed to prevent proper pairing of its wild-type homolog, thus inducing it to autogenously silence itself (Shiu *et al.* 2001). The MSUD testers *pan-2*; *his-3*::*his-3^+^ Bml^r^ A* (FGSC 8755); *pan-2*; *his-3*::*his-3^+^ Bml^r^ a* (FGSC 8756); *pan-2*; *his-3*::*his-3^+^ mei-3^+^ A* (FGSC 8759); and *pan-2*; *his-3*::*his-3^+^ mei-3^+^ a* (FGSC 8760) (hereafter designated as :: *Bml^r^ A*, :: *Bml^r^ a*, ::*mei-3 A*, and ::*mei-3 a*) are described by Raju *et al.* (2007). Another tester, *rid his-3*; *VIIL*::*r^ef2^-hph A* (ISU3117) (hereafter ::*r^ef2^* ), was a gift from Dr. Tom Hammond (Illinois State University). The ::*Bml^r^* and ::*mei-3* testers have an extra copy of the *bml* (β-tubulin) or *mei-3* gene inserted ectopically in the *his-3* locus in chromosome 1, whereas the tester strain ::*r^ef2^* has a copy of the *r* (*round spores*) gene inserted ectopically into chromosome 7. In a cross of the tester with wild-type, the ectopic copy remains unpaired in meiosis and results in elimination of all its homologous transcripts, including from the paired endogenous copies. In crosses of ::*Bml^r^*, ::*mei-3*, and ::*r^ef2^* with the wild-type, the *bml*, *mei-3*, and *r* genes, respectively, are silenced. Silencing of the *bml* or *mei-3* gene arrests normal ascus development (Raju *et al.* 2007; Kasbekar *et al.* 2011), and silencing of *r* causes all eight ascospores to be round instead of having the normal spindle shape. Homozygous *tester A* × *tester a* crosses do not show MSUD, nor do crosses of the testers with the *Sad-1* suppressor of MSUD, and the asci developed normally (Raju *et al.* 2007; Kasbekar *et al.* 2011).

*N. tetrasperma* has standard strains 85 *A* (FGSC 1270) and 85 *a* (FGSC 1271); the *E* mutants are *lwn*; *al(102)*, *E A* (FGSC 2783) and *lwn*; *al(102)*, *E a* (FGSC 2784) (hereafter *E A* and *E a*). *N. crassa*/*N. tetrasperma* hybrid strain is *C4,T4 a* (FGSC 1778). The *C4,T4 a* strain has four *N. crassa* great-grandparents and four *N. tetrasperma* great-grandparents (Metzenberg and Ahlgren 1969). The *N. crassa* great-grandparents were of the OR background, whereas the *N. tetrasperma* great-grandparents were of the 343.6 *A E* background (Metzenberg and Ahlgren 1969).

Neurospora genetic analysis was performed essentially as described by Davis and De Serres (1970). The alternative recipe of Metzenberg (2003) was used for making Medium N.

### Oligonucleotide primers used for PCR

Supporting Information, Table S1 lists the sequences of the oligonucleotide primers used to test for the translocation breakpoint junctions and to amplify sequences of the *ad-7 RIP3C* and *RIP3T* alleles.

### Outline of the introgression crosses and characterization of the resultant strains

Crosses between *N. crassa* and *N. tetrasperma* strains are almost completely sterile. However, both *N. crassa* strain OR *A* and *N. tetrasperma* strain 85 *A* can cross with the *N. crassa*/*N. tetrasperma* hybrid strain *C4,T4 a* and produce viable progeny (Perkins 1991; also see Table 3 of this article); therefore, we used the *C4,T4 a* strain as a bridging strain for the initial introgression crosses. The *N. crassa T* strains were crossed with *C4T4 a* and *T* progeny from these crosses (designated *T^1xC4T4^*) were distinguished from their *Dp* and *N* siblings by PCR with breakpoint junction-specific primers. Nominally, 50% of the genome of *T^1xC4T4^* progeny is derived from the *C4,T4 a* parent. The *T^1xC4T4^A* strains were crossed with *C4,T4 a* to obtain the *T^2xC4T4^* progeny in a similar manner. Crosses of *T^2xC4T4^* with the opposite mating-type derivative of strain *85* were productive, and their *T* progeny were designated as *T^1x85^*. Likewise, *T^1x85^* × *85* yielded *T^2x85^*, etc. After two to three iterations of the crosses with *85*, we recovered progeny ascospores that produced mycelium of dual mating specificity characteristic of *N. tetrasperma*. That is, the resulting mycelium could cross with both 85*A* and *a*, and it could also undergo a self-cross. A heterokaryotic strain containing all three breakpoints (A, B, and C) is potentially of genotype [*T* + *N*] or [*Dp* + *Df*].

The [*T* + *N*] and [*Dp* + *Df*] heterokaryons are distinguishable, because the former produces homokaryotic conidial derivatives of both mating types, whereas the latter produces viable homokaryons of only the mating type of the *Dp* nucleus. Conidia from self-fertile heterokaryotic strains were streaked onto Vogel’s FGS medium, and well-isolated conidial germlings were transferred to SCM to distinguish self-fertile (heterokaryotic) from self-sterile (homokaryotic) conidial derivatives. The mating type of the self-sterile conidial derivatives was determined by crossing to the single mating type derivatives *85 a* and *85 A*. If all the self-sterile conidial derivatives are of a single mating type, then the heterokaryon from which they were derived is likely [*Dp* + *Df*], else it is [*T* + *N*]. The results were confirmed by PCR with primers for the breakpoint junctions and *mat* ideomorphs and DNA of the homokaryotic conidial derivatives.

Four homokaryotic *T*-type conidial derivatives from the self-fertile [*T* + *N*] heterokaryons, namely, *T(EB4)^Nt^ a* from the heterokaryon 3E1 (Table 2 serial number 3), *T(IBj5)^Nt^ a* from I4 (Table 2 serial number 18), *T(UK14-1)^Nt^ a* from U9 (Table 2 serial number 24), and *T(B362i)^Nt^A* from 19B7 (Table 2 serial number 35), were used in the experiments and results are summarized in Table 3 and Table 4.

### A note on strain nomenclature

**Figure 2:**
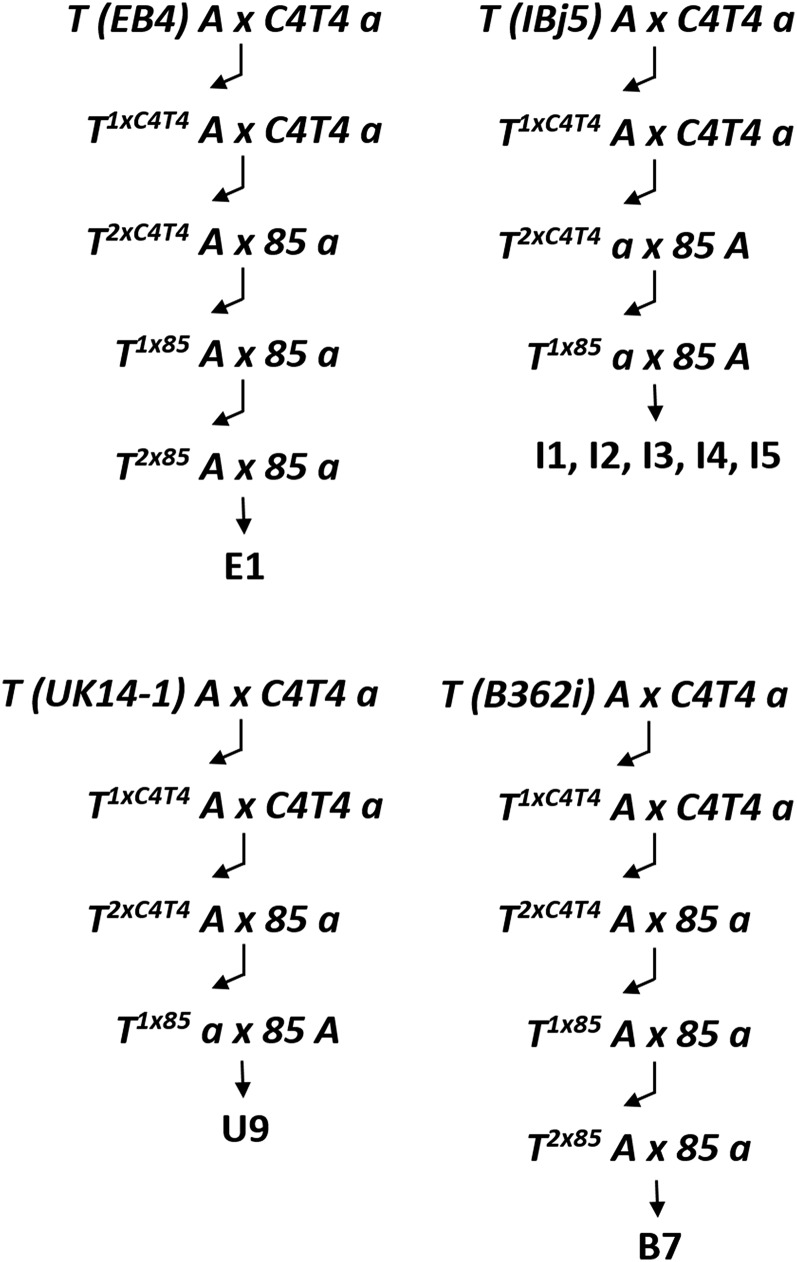
Introgression crosses. *T(EB4) A*, *T(IBj5) A*, *T(UK14-1) A*, and *T(B362i) A* strains of *N. crassa* were crossed with the *C4T4 a* hybrid strain. Bent arrows represent PCR with breakpoint junction-specific primers to distinguish the translocation progeny (*e.g.*, *T^1xC4T4^*) from their *Dp* and *N* siblings. *T^1xC4T4^A* × *C4T4 a* yielded *T^2xC4T4^ A* or *T^2xC4T4^ a* strains, which were productive in crosses with opposite mating-type homokaryotic derivatives of *N. tetrasperma* strain *85*. *T^1x85^* progeny were crossed with *85 a* or *85 A* to obtain the self-fertile heterokaryotic strains I1-I5 (for *IBj5*) and U9 (for *UK14-1*), or the *T^2x85^* strains (for *EB4* and *B362i*). Crosses of *T^2x85^* with *85 a* produced the heterokaryons E1 and B7. From self-cross of the heterokaryons, we obtained self-fertile progeny that were genotyped as [*T* + *N*] or [*Dp* + *Df*] (see Table 2).

The different self-fertile strains listed in Table 2 were named using the following rules. The letters E, I, U, or B in the name identify strains derived from introgressions of, respectively, the *N. crassa* translocations *T(EB4)*, *T(IBj5)*, *T(UK14-1)*, and *T(B362i)*. The strains E1, U9, B7, and I1-I5 were self-fertile heterokaryons obtained from the cross of homokaryotic *T(EB4)*, *T(UK14-1)*, *T(B362i)*, and *T(IBj5)* strains with either *85 a* or *85 A* (see Figure 2). Strains 1E1, 2E1, 3E1, etc., were derived from the self-cross of strain E1; likewise, strains 1I1, 2I1, 3I1, etc., were from the self-cross of strain I1; 1U9, 2U9, 3U9, etc., were from the self-cross of strain U9; and 11B7, 18B7, and 19B7 were from self-crosses of strain B7. Further, strains 1(1U9), 2(1U9), 3(1U9), etc., were from self-cross of strain 1U9; 6(19B7) was from self-cross of strain 19B7; and 1[6(19B7)], 2[6(19B7)], etc., were from the self-cross of strain 6(19B7).

## Results

### *N. tetrasperma* [*T* + *N*] and [*Dp* + *Df*] strains can switch genotype via self-crosses

The salient features of the *N. crassa* translocations *T(EB4)*, *T(IBj5)*, *T(UK14-1)*, and *T(B362i)* are summarized in Table 1, along with the accession numbers of their breakpoint junction sequences. The introgression of these translocations into *N. tetrasperma* is outlined in the *Materials and Methods* section, and Figure 2 schematically presents the actual crosses performed.

**Table 1:** Translocations used in this study

Translocation	Size (bp)	Genes (N)	Breakpoint Junction Sequence (Accession Number)		
			A	B	C
*T(VR > VII) EB4*	145,282	39	GQ504681	GQ504682	GQ504683
*T(VIL > IR) IBj5*	405,319	120	GQ504684	GD504685*^a^*	—
*T(VIR > VL) UK14-1*	490,958	126	GQ504703	—	NA
*T(IVR > IL) B362i*	118,782	36	GQ504697	GQ504698	GQ504699

NA, not applicable.

a *T(IBj5)* may be a complex rearrangement (see text).

Introgression of *T(EB4)* yielded the self-fertile heterokaryotic strain designated E1 (Figure 2). Using E1 genomic DNA as template and *T(EB4)* breakpoint junction-specific primers, all three breakpoint junctions (A, B, and C) of *T(EB4)* could be amplified by PCR (data not shown). A heterokaryon possessing all the three breakpoints is potentially of genotype [*T(EB4)* + *N*] or [*Dp(EB4)* + *Df(EB4)*]. Heterokaryons of genotype [*T(EB4)* + *T(EB4)*], [*T(EB4)* + *Dp(EB4)*], or [*T(EB4)* + *Df(EB4)*] also fulfill this criterion, but, they were deemed to be less likely because one or more crossover is required to generate them. Eight self-fertile progeny from the self-cross of E1 were analyzed and the results, which are summarized in Table 2 (serial numbers 1–8), established that progeny 2E1, 4E1, and 6E1 were of the [*T(EB4)* + *N* ] genotype, whereas 1E1, 9E1, and 12E1 were [*Dp(EB4)* + *Df(EB4)*]. Only six self-sterile conidial derivatives were obtained for 3E1, and PCR revealed all to be type *T(EB4) a*, suggesting that 3E1 has the genotype [*T(EB4) a* + *Df(EB4) A*]. However, given the small numbers tested, and given the possibility of skewed segregation, our results do not exclude the [*T(EB4) a* + *N A*] genotype. No self-sterile conidial derivatives were obtained from 13E1; therefore, its genotype was not determined.

**Table 2:** Genotype of self-fertile strains

Serial No.	Self-Fertile Strain	Conidial Derivatives (N)	Self-Sterile Derivatives (N)	*mat* *a* (N)	*mat A* (N)	Strain Genotype Indicated by PCR
1	1E1	67	25	0	25	[*Df a* + *Dp A*]
2	2E1	38	10	5	5	[*N a* + *T A*]
3	3E1	28	6	6	0	[*T a* + *Df A*]*^a^*
4	4E1	85	12	1	11	[*T a* + *N A*]
5	6E1	129	30	10	20	[*T a* + *N A*]
6	9E1	62	14	0	14	[*Df a* + *Dp A*]
7	12E1	49	16	16	0	[*Dp a* + *Df A*]
8	13E1	16	0	0	0	ND
9	1(6E1)	59	9	4	5	ND
10	3(6E1)	63	7	0	7	[*Df a* + *Dp A*]
11	4(6E1)	54	10	4	6	ND
12	1(9E1)	40	8	6	2	[*T a* + *N A*]
13	2(9E1)	56	10	0	10	ND
14	3(9E1)	65	11	8	3	ND
15	I1	87	17	8	0	[*Dp a* + *Df A*]
16	I2	75	6	0	6	[*Df a* + *N A*]*^b^*
17	I3	27	5	0	5	[*Df a* + *T A*]*^c^*
18	I4	121	34	2	8	[*T a* + *N A*]
19	I5	62	23	4	0	[*Dp a + Df A*]
20	1I1	50	4	0	4	[*T a* + *N A*]*^d^*
21	2I1	24	16	16	0	[*Dp a* + *Df A*]
22	3I1	60	9	2	7	[*T a* + *N A* + *Df A*]*^e^*
23	4I1	26	0	0	0	ND
24	1I4	30	1	0	1	[? *a* + *T A*]*^f^*
25	2I4	30	18	9	9	[*T a* + *N A*]
26	U9	24	5	1	4	[*T a* + *N A*]
27	1U9	19	8	8	0	[*Dp a* + *Df A*]
28	2U9	49	9	1	8	ND
29	3U9	56	7	5	2	ND
30	4U9	80	0	0	0	ND
31	5U9	44	8	3	5	[*N a* + *TA*]
32	1(1U9)	0	0	0	0	ND
33	2(1U9)	45	12	3	9	[*N a* + *T A*]
34	3(1U9)	10	10	0	10	[*Df a* + *Dp A*]
35	B7	10	10	10	0	[*Dp a* + *Df A*]
36	11B7	30	30	30	0	[*Dp a* + *Df A*]
37	18B7	20	20	20	0	[*Dp a* + *Df A*]
38	19B7	11	8	3	5	[*N a* + *T A*]
39	24B7	7	7	7	0	[*Dp a* + *Df A*]
40	28B7	33	33	33	0	[*Dp a* + *Df A*]
41	30B7	6	6	6	0	[*Dp a* + *Df A*]
42	31B7	0	0	0	0	ND
43	6(19B7)	11	11	11	0	[*Dp a + Df A*]
44	1[6(19B7)]	4	3	2	1	[*N a + Dp A*]
45	2[6(19B7)]	9	7	2	5	[*N a + T A*]
46	4[6(19B7)]	24	1	0	1	[? *a + T A*]*^h^*
47	5[6(19B7)]	15	14*^g^*	12	2	[*N a + T A*]

ND, not determined.

a,b [*T a* + *N A*] and *^c^*[*N a* + *T A*] genotypes are not excluded, see text.

d Genotype inferred from the fact that all four self-sterile conidial derivatives were *mat A* and failed to amplify any of the *T(IBj5)* junction fragments, but control PCRs performed with DNA from “sibling” self-fertile conidial derivatives amplified both the A and B junction fragments, thus establishing that the *mat a* nucleus must be type *T*.

e DNA from two of the seven *mat-A* conidial derivatives amplified the A junction, but DNA from the other five did not. See text for details.

f Only one self-sterile conidial derivative was obtained. It was *mat A* and its DNA amplified the A and B junction fragments of *T(IBj5)*, thus establishing it as type *T*. The *mat a* nucleus could be type *N*, *T*, *Dp*, or *Df*. Compare with footnote *d*.

g All 15 conidial derivatives were self-sterile, but PCR showed that 14 were homokaryotic for *mat A* or *mat a*, and one was a [*mat a* + *mat A*] heterokaryon. Because the 5[6(19B7)] strain was self-fertile, the self-sterility of the heterokaryotic conidial derivative can be attributed to homozygosity for a secondary mutation.

h Only one self-sterile conidial derivative was obtained. It was *mat A* and its DNA amplified the A, B, and C junction fragments of *T(B362i)*, thus establishing it as type *T*. The *mat a* nucleus could be type *N*, *T*, *Dp*, or *Df*. Compare with footnotes *d* and *f*.

From self-crosses of strains 6E1 and 9E1 (see above), we examined 39 and 24 progeny and found that 12 and 9, respectively, were self-fertile. From a subset of self-fertile progeny, we obtained self-sterile (*i.e.*, homokaryotic) conidial derivatives and determined their mating type by crossing with 85 *a* and 85 *A*. Of three self-fertile progeny tested from strain 6E1, two appeared to be [*T(EB4) a* + *N A*] and one was [*Dp(EB4) a* + *Df(EB4) A*]; of three self-fertile progeny tested from strain 9E1, one appeared to be [*Dp(EB4) a* + *Df(EB4) A*] and the other two appeared to be [*T(EB4) a* + *N A*]. We used PCR to confirm the genotype of the [*Dp(EB4) a* + *Df(EB4) A*] progeny of 6E1, and the genotype of one [*T(EB4) a* + *N A*] progeny of 9E1 (Table 2, serial numbers 10 and 12). These results showed that self-crosses of both [*T(EB4)* + *N*] and [*Dp(EB4)* + *Df(EB4)*] heterokaryons can again generate [*T(EB4)* + *N*] and [*Dp(EB4)* + *Df(EB4)*] progeny.

The translocations *T(IBj5)*, *T(UK14-1)*, and *T(B362i)* were introgressed in a similar manner (Figure 1), and we recovered heterokaryons of genotypes [*T(IBj5)* + *N*] and [*Dp(IBj5)* + *Df(IBj5)*] (Table 2, serial numbers 15, 18-21, and 25), [*T(UK14-1)* + *N*] and [*Dp(UK14-1)* + *Df(UK14-1)*] (Table 2, serial number 26, 27, 31, 33, and 34), and [*T(B362i)* + *N*] and [*Dp(B362i)* + *Df(B362i)*] (Table 2, serial numbers 35-41, 43, 45, and 47). Again, self-crosses of the [*T* + *N*] and [*Dp* + *Df*] heterokaryons produced progeny of the alternative genotype. Specifically, self-cross of the [*Dp(IBj5)* + *Df(IBj5)*] type strain I1 produced the [*T(IBj5)* + *N*] type progeny strain 1I1 (Table 2, serial numbers 15 and 20); of the [*T(UK14-1)* + *N*] type strain, U9 produced the [*Dp(UK14-1)* + *Df(UK14-1)*] type progeny strain 1U9, whose self-cross, in turn, produced the [*T(UK14-1)* + *N*] type strain 2(1U9) (Table 2, serial numbers 26, 27, and 33); and of the [*Dp(B362i)* + *Df(B362i)*] type strain, B7 produced the [*T(B362i5)* + *N*] type progeny strain 19B7, whose self-cross, in turn, produced the [*Dp(B362i)* + *Df(B362i)*] type progeny strain 6(19B7), whose self-cross produced the [*T(B362i5)* + *N*] type progeny 2[6(19B7)] and 5[6(19B7)] (Table 2, serial numbers 35, 38, 43, 45 and 47). In sum, our results show that [*T* + *N*] and [*Dp* + *Df*] genotypes can be interchanged through self-crosses.

The genotype of two heterokaryons was found to be putatively [*Df(IBj5) a* + *N A*] and [*Df(IBj5) a* + *T(IBj5)A*] (Table 2, serial numbers 16 and 17), but our results do not rule out the possibility that skewed segregation in small numbers might account for the absence of self-sterile *T(IBj5)* or *N* conidial types, respectively, from what in fact might be [*T(IBj5)* + *N*] heterokaryons. One heterokaryon was found to contain three nuclear types, and its genotype was [*T(IBj5) a* + *NA* + *Df(IBj5)A*] (Table 2, serial number 22). It got flagged because two of the seven *mat A* self-sterile conidial derivatives examined possessed only the A, but not B, junction of *T(IBj5)*, whereas the other five did not possess either junction. A homokaryon bearing only the A junction is not expected to be viable because it contains an uncomplemented *Df* chromosome; therefore, we presume the two self-sterile *mat A* derivatives were [*NA* + *Df(IBj5)A*] heterokaryons and we infer that the genotype of the self-fertile strain was [*T(IBj5) a* + *NA* + *Df(IBj5)A*]. We defer to the *Discussion* section a consideration of how such a strain might have arisen.

### [*Dp(B362i)* + *Df(B362i)*] heterokaryons yield only self-sterile conidial derivatives

The search for self-sterile homokaryotic conidial derivatives (above) was expected to inevitably also identify self-fertile ones. A majority of conidial derivatives from both [*T* + *N*] and [*Dp* + *Df*] strains of *EB4*, *IBj5*, and *UK14-1* were self-fertile (Table 2, serial numbers 1-33), and self-fertile conidial derivatives (or derivatives heterokaryotic for mating type) were also obtained from [*T(B362i)* + *N*] (Table 2, serial numbers 38, 45, and 47). Therefore, we were very surprised to find that all the 117 conidial derivatives examined from seven different [*Dp(B362i)* + *Df(B362i)*] heterokaryons were self-sterile homokaryons (Table 2, serial numbers 35–37, 39–41, and 43).

As an additional control we performed the cross *Dp(B362i) a* × *85 A* and tested 61 progeny, of which 39 proved to be self-fertile (*i.e.*, heterokaryotic). We examined 10 to 25 conidial derivatives from each of 16 self-fertile progeny, and in every case we found some self-fertile conidial derivatives. Additionally, for three progeny, we also identified self-sterile conidial derivatives of both mating types, and by PCR we could establish that the genotype of one progeny was [*Dp(B362i) a* + *Dp(B362i) A +N A*], and the other two were [*Dp(B362i) a* + *Dp(B362i) A*]. Thus, unlike their [*Dp(B362i) a* + *Df(B362i) A*] counterparts, the [*Dp(B362i)* + *N*] heterokaryons were able to make self-fertile heterokaryotic conidia. Additionally, among the progeny from the self-cross of a [*Dp(B362i) a* + *Df(B362i) A*] self-fertile heterokaryon, we recovered one that was [*N a + Dp(B362i) A*] type (Table 2, serial number 44), and of four conidial derivatives examined, one was self-fertile. Therefore, the absence of self-fertile conidia from [*Dp(B362i) a* + *Df(B362i) A*] heterokaryons appears to be exceptional. The implication of this phenotype is considered in the *Discussion* section.

### Characterizing the *T*-type homokaryons

The homokaryotic *T*-type conidial derivatives designated as *T(EB4)^Nt^* , *T(IBj5)^Nt^*, *T(UK14-1)^Nt^*, and *T(B362i)^Nt^* were obtained from the self-fertile [*T* + *N*] heterokaryons (see *Materials and Methods*) and were found to behave like *bona fide* *N. tetrasperma* strains. That is, their crosses with opposite mating-type derivatives of *N. tetrasperma* strain *85* were fertile, whereas their crosses with *N. crassa* OR strains of opposite mating type were as infertile as an OR × *85* interspecies cross (Table 3). Control crosses of the *N. crassa T* strains (*T^Nc^* ) with the OR strains of the opposite mating type were productive, but the crosses of the *T^Nc^* with the opposite mating type derivatives of strain *85* were sterile (Table 3). The *C4,T4 a* hybrid strain produced viable ascospores in crosses with both OR *A* and *85 A* (Table 3).

**Table 3:** Ascospore productivity in crosses of *T* strains with OR and 85

Strain*^a^*	Productivity in Cross	
	× OR	× 85
*T (EB4)^Nc^*	++++	—
*T (IBj5)^Nc^*	++++	—
*T (UK14-1)^Nc^*	++++	—
*T (B362i)^Nc^*	++++	—
*T (EB4)^Nt^*	—	++++
*T (IBj5)^Nt^*	—	++++
*T (UK14-1)^Nt^*	—	++++
*T (B362i)^Nt^*	—	++++
OR *A*	++++	—
85 *A*	—	++++
*C4,T4 a*	+++	++

Ascospore yield: − <100; 100 < ++ < 500; 500 < +++ < 5000; ++++ > 5000.

a *T^Nc^* are *N. crassa T* strains and *T^Nt^* are the translocation breakpoint-bearing homokaryons derived from self-fertile heterokaryons.

To obtain larger numbers of eight-spored asci, we crossed the *T^Nt^* strains with *E* strains of the opposite mating type. The *T(EB4)^Nt^ a* × *E A* and *T(UK14-1)^Nt^ A* × *E a* crosses were productive and produced 8:0 and 4:4 ascus types at comparable frequencies (Table 4), which is a characteristic of *IT* × *N* and *QT* × *N* crosses in *N. crassa*. Surprisingly, the crosses *T(IBj5)^Nt^ a* × *E A* and *T(B362i)^Nt^ A* × *E a* did not produce any 8:0 or 6:2 asci, and most ascospores from these crosses (∼90 and 70%, respectively) were white and inviable (Table 4). However, the crosses *T(IBj5)^Nt^A* × *85 a* and *T*(*B362i*)*^Nt^A* × *85 a* produced a few eight-spored asci, and in them the 8:0 and 4:4 frequencies appeared to be comparable (Table 4).

**Table 4:** Ascus (octets) types from *T* × *N* crosses in *N. crassa* (*Nc*) and *N. tetrasperma* (*Nt*)

Cross	Octets (N)	Ascus Type (%)				
		8:0	6:2	4:4	2:6	0:8
***T ^Nc^* × OR *a***						
*T (EB4)^Nc^ A*	56	56	5	36	3	0
*T* (*IBj5*)*^Nc^ A*	72	31	43	19	6	1
*T* (*UK14-1*)*^Nc^ A*	86	21	46	24	7	4
*T* (*B362i*)*^Nc^ A*	83	55	13	32	0	0
***T ^Nt^* × *E***						
*T* (*EB4*)*^Nt^ a*	96	32	29	40	2	0
*T* (*IBj5*)*^Nt^ a*	159	0	0	6	27	67
*T* (*IBj5*)*^Nt^ A* × 85 *a*	275*^a^*	3	2	1	0	0
*T* (*UK14-1*)*^Nt^ A*	101	47	15	37	1	1
*T* (*B362i*)*^Nt^ A*	145	0	0	49	19	32
*T* (*B362i*)*^Nt^ A* × 85 *a*	124*^b^*	1	0	2	0	0

a Asci (n = 275) were collected on water agar and the fraction of four-, five-, six-, and seven-spored asci were, respectively, 58%, 19%, 12%, and 4%. Shown here is the distribution of ascus type among the 6% that were eight-spored.

b Asci (n = 124) were collected on water agar and the fraction of four-, five-, six-, and seven-spored asci were, respectively, 56%, 25%, 15%, and 2%. Shown here is the distribution of ascus type among the 3% that were eight-spored.

Results summarized in Table 5 show that the crosses of *T*(*EB4*)*^Nt^a* and *T*(*IBj5*)*^Nt^a* with *T*(*UK14-1*)*^Nt^A* and *T*(*B362i*)*^Nt^A* produced many four-spored asci, consistent with the conclusion that the *T^Nt^* strains behave like *bona fide* *N. tetrasperma* strains. However, the crosses also produced many nonfour spored asci, probably because the *T^Nt^* strains still retain a significant fraction of the ancestral non-*85* genetic background (*N. crassa T* or *C4T4 a*). All ascospores produced in the cross *T* (*IBj5*)*^Nt^ a* × *T* (*B362i*)*^Nt^ A* were white and inviable. A model to explain this result is considered in the *Discussion* section.

**Table 5:** Ascus types from *N. tetrasperma T* × *T* crosses

Cross	Asci (N)	Ascus Type (%)								
		4	5	6	7	8:0	6:2	4:4	2:6	0:8
*T* (*EB4*)*^Nt^ a* × *T* (*UK14-1) ^Nt^ A*	101	47	28	14	9	1	0	2	0	0
*T* (*EB4*)*^Nt^ a* × *T* (*B362i*)*^Nt^ A*	118	24	29	20	13	0	1	8	1	4
*T* (*IBj5*)*^Nt^ a* × *T* (*UK14-1*)*^Nt^ A*	144	72	22	4	2	0	0	0	0	0
*T* (*IBj5*)*^Nt^ a* × *T* (*B362i*)*^Nt^ A*	149	28	33	26	10	0	0	0	0	3

Asci were collected on water agar and the fraction of four-, five-, six-, seven-, and eight-spored asci was scored. The eight-spored asci are classified based on number of black:white ascospores. All the ascospores from *T* (*IBj5*)*^Nt^ a* × *T* (*B362i*)*^Nt^ A* were white and inviable.

### RIP in a *Dp*-heterozygous *N. tetrasperma* cross

In *N. crassa*, crosses involving *Dp* strains can generate RIP-induced mutant progeny (Perkins *et al.* 1997). Therefore, we expected crosses of *Dp* strains in *N. tetrasperma* also to yield RIP-induced mutants. *Dp(EB4)* duplicates the *ad-7* (*adenine requiring-7*) gene (Perkins 1997). Ascospores from *Dp(EB4) a* × *E A* were germinated on adenine-supplemented Vogel’s FGS medium, 130 germlings were picked to adenine-supplemented Vogel’s glucose medium, and then their growth was tested on unsupplemented Vogel’s glucose medium. Three adenine-requiring auxotrophic strains were identified among 125 progeny examined. Two (RIP1 and RIP2) were *N*-type homokaryons; their *ad-7* locus, derived from strain *85 A*, was found to be altered by several RIP mutations (G:C to A:T transitions) (Table 6). The third auxotrophic strain (RIP3) was a heterokaryon for mating type, showing that *E* has incomplete penetrance. Both the *mat A* and *mat a* nuclei of this heterokaryon must contain RIP-induced mutant *ad-7* alleles, and its genotype potentially can be [*Dp* + *N*], [*Dp* + *Dp*], or [*N* + *N*], with the latter two resulting from second-division segregation. Both copies of the 145-kb duplicated segment that participated in RIP are “captured” in the [*Dp* + *N*] and [*Dp* + *Dp*] types. The *T* recipient chromosome has *N. crassa*–derived sequences, whereas the *N* homolog of the *T* donor chromosome has 85 *A*-derived sequences; therefore, their *ad-7* mutant alleles, *RIP3C* and *RIP3T*, are distinguishable. Both *RIP3C* and *RIP3T* showed evidence of RIP (Table 6). Interestingly, all the *RIP3T* mutations were G to A changes, whereas those in *RIP3C* were C to T changes.

**Table 6:** RIP-induced *ad-7* mutants from *Dp(EB4)* × *E*

	*Nt*	*Nc*	*RIP1**^b^*	*RIP2**^c^*	*RIP3T**^d^*
*85 A**^a^*	6 (2)	69 (42)	45 (6)	40 (6)	11
*RIP3C**^e^*	—	4	—	—	—

Comparison of *ad-7* gene sequences from 85 *A* (*N. tetrasperma* FGSC 1270 KP006652); *Nt* (*N. tetrasperma* FGSC 2508 sequence ID GL891302, nucleotides 1022253 to 1024228); *Nc* (*N. crassa* OR74A FungiDB database (fungidb.org) NCU04216, nucleotides 5238499 to 5240480); and the RIP-induced mutant alleles *RIP1* (KP006653), *RIP2* (KP006654), *RIP3C* (KR349720), and *RIP3T* (KR349721). The *RIP3C* sequence corresponds to nucleotides 5238499 to 5240184 of the *Nc* sequence and *RIP3T* to nucleotides 1022550 to 1024228 of the *Nt* sequence. Numbers in parenthesis indicate nucleotide differences in the introns, and altered amino acid residues are listed below in bold. Superscripts indicate accession numbers of sequences determined in this work.

85 *A* /*Nt* (4): T40T, S216S, I306I, F475F.

85 *A*/*Nc* (27): T40T, F94F, S132S, D156D, D189D, R201R, S216S, L221L, F229F, L233L, G245G, Y264Y, I306I, D377D, A396A, A404A, A421A, P423P, V445V, A456A, P462P, F475F, G506G, T522T, **E524D**, I538I, H541H.

85 *A*/*RIP1* (39): **H22Y**, N97N, **H106Y**, I111I, **L120F**, Q122*, T130T, D131D, N139N, **R222H**, **H249Y**, Q250*, A268A, **P270T**, T272T, I273I, S391S, A396A, R399R, I402I, A404A, P409P, I410I, I411I, A421A, **P423L**, Q424*, L426L, **H429Y**, D435D, H439H, I440I, Q448*, **H464Y**, Q465*, T507T, N508N, V518V, **H541Y.**

85 *A*/*RIP2* (34): Q49*, Y72Y, N97N, G101G, I111I, **L120F**, **H127Y**, V153V, S203S, Y212Y, Q241*, Q245*, Q250*, Q254*, A291A, **L298F**, V303V, I309I, T315T, **S324L**, V341V, F345F, I346I, A363A, G383G, T384T, A396A, R399R, **H414Y**, N534N, **H541Y**, N542N, S546S.

85 *A*/*RIP3T* (11): D133N, L136L, V153I, D157N, **A161T,** E167E, **R168K,** V175I, **A177T, G181S, G192S.**

*Nc*/*RIP3C* (4): V175V, Q247*, V445V, Q448*.

a KP006652.

b KP006653.

c KP006654.

d KR349720.

e KR349721.

### Crosses involving *C4,T4* a and *85 A/a* show relatively weak MSUD

Ramakrishnan *et al.* (2011) classified wild-isolated *N. crassa* strains into three types based on the strength of MSUD in their crosses with tester strains, namely, “OR” (which includes the standard laboratory strains OR *A* and OR *a*), “Sad” (which includes the semi-dominant *Sad* suppressors of MSUD obtained in the OR background), and “Esm.” MSUD was strongest in crosses with the OR type, of intermediate strength in crosses with the Esm type, and weakest in crosses with the Sad type. We performed crosses of *C4,T4 a* with the MSUD tester strains. The results summarized in Figure S1 show that, like the Esm-type strains, *C4,T4 a* also partially suppresses MSUD. The Esm phenotype of *C4,T4 a* must have come from its 343.6 *A E* ancestor.

In *N. crassa*, crosses of *Dp(EB4)* and *Dp(IBj5)*strains with the OR-type strains were barren, whereas their crosses with the Sad-type strains were fertile (Ramakrishnan *et al.* 2011). Most Esm-type strains gave a fertile cross with *Dp(EB4)* and gave a barren cross with *Dp(IBj5)*. Some Esm-type strains gave barren crosses with both the *Dp*s, and fewer still gave fertile crosses with both the *Dp*s. Other results of Ramakrishnan *et al.* (2011) suggested that *N. tetrasperma* strain 85 is the Esm type. We crossed the *N. tetrasperma Dp(EB4)* and *Dp(IBj5)* strains with opposite mating-type derivatives of strain 85 and found the crosses were as productive as crosses of *T(EB4)* and *T(IBj5)* with strain 85 (data not shown). This is consistent with the classification of strain 85 as the Esm type or the Sad type.

## Discussion

We have made self-fertile heterokaryotic [*T* + *N*] and [*Dp* + *Df*] Neurospora strains by introgressing translocations from *N. crassa* into *N. tetrasperma*. Both types of heterokaryons when self-crossed again produced [*T* + *N*] and [*Dp* + *Df*] progeny. [*T* + *N*] and [*Dp* + *Df*] mycelia were distinguishable because the former yielded self-sterile homokaryotic conidial derivatives of both mating types, whereas the latter produced homokaryons of only the mating type of the *Dp* component. The introgressions depended on the unambiguous identification, by PCR with breakpoint junction-specific primers, of the translocation (*T*) progeny from each round of crosses, as represented by the bent arrows in Figure 2. In 1984, N. B. Raju crossed a *N. crassa* rearrangement strain with the giant-spore *Banana* (*Ban*) mutant with the hope that he could rescue the [*Dp* + *Df*] heterokaryon. He had previously used this approach to rescue *Sk^S^* nuclei from a *Sk^S^* × *Sk^K^* cross in [*Sk^S^* + *Sk^K^*] heterokaryotic ascospores (Raju 1979); however, analysis of progeny nuclei in the mixed cultures from the later experiment was not easy, and his efforts were inconclusive (N. B. Raju, personal communication). D. D. Perkins also alluded to his obtaining [*Dp* + *Df*] heterokaryons (Perkins 1997), but his results remained unpublished. Therefore, to the best of our knowledge, this is the first report of heterokaryons with complementary duplications and deficiencies in their constituent nuclei.

The introgressions were facilitated by use of the *C4,T4 a* bridging strain (Perkins 1991). We found that *C4,T4 a* is a moderate suppressor of MSUD (Supporting Information, Figure S1). Shiu *et al.* (2001) had shown that the *N. crassa* MSUD suppressor *Sad-1* can partially breach the *N. crassa*/*N. tetrasperma* interspecies barrier. Although *C4,T4 a* does not suppress MSUD as strongly as *Sad-1 a*, its moderate suppressor phenotype might contribute to increase ascospore production in crosses with both *N. crassa* and *N. tetrasperma* strains. The *N. crassa T* strains were first crossed with *C4T4 a*, and *T A* progeny from this cross were crossed with *C4T4 a*. *T* progeny from the latter crosses were used to initiate two or three additional rounds of crosses with opposite mating-type derivatives of *N. tetrasperma* strain *85*. Although this sequence of introgression crosses can be indefinitely continued, we stopped once they began yielding self-fertile progeny. Nominally, one nucleus in the B7, E1, I1, I2, I3, I4, I5, and U9 self-fertile progeny of Figure 1 derives one-eighth or one-fourth of its genome from the non-*85* genetic background (*N. crassa T* or *C4T4 a*) and, depending on the translocation, this fraction is enriched for sequences from the translocation chromosomes (1, 4, 5, 6, or 7). Therefore, in principle, by sequencing one should be able to identify strain *85* genome segments that are replaceable by non-*85* sequences without losing the pseudohomothallic life cycle.

Self-crosses of the [*T* + *N*] / [*Dp* + *Df*] strains also appeared to produce [*T* + *Df* ], [*N* + *Dp*], and [*N* + *Df*] genotypes. These genotypes can be generated by a breakpoint-proximal crossover on the *T* donor or recipient chromosome, followed by second-division segregation of the resulting *Df*-bearing or *N*-bearing chromosome into both *mat a* and *mat A* nuclei. Breakpoint-proximal crossover in *IT* × *N* and *QT* × *N* crosses in *N. crassa* and in *IT* × *E* and *QT* × *E* crosses in *N. tetrasperma* produces 6:2 ascus types that have two ascospores each of *T*, *N*, *Dp*, and *Df* types (see Figure 1 in Perkins 1997). One self-fertile heterokaryon had the genotype [*T a* + *NA* + *Df A*]. This triple heterokaryon could have arisen from an interstitial crossover; that in *N. crassa* would have produced a 6:2 ascus with two each of the *T a*, *N A*, *Dp a*, and *Df A* nuclei. Presumably, during ascus development, three nuclei (*T a*, *N A*, and *Df A*) instead of two were partitioned into the ascospore from which this strain was derived. In *N. crassa* crosses heterozygous for the *Fsp-1* (*Four-spore-1*) or *Fsp-2* (*Four-spore-2*) mutant, rare two-spored and three-spored asci are occasionally formed that contain heterokaryotic ascospores (Raju 1986; Perkins *et al.* 2001). Our [*T a* + *NA* + *Df A*] strain may have had a similar provenance.

The 8:0 and 4:4 ascus types in *IT* × *N* crosses in *N. crassa*, and now among the eight-spored asci from *IT* × *N* crosses in *N. tetrasperma*, arise, respectively, from alternate and adjacent 1 segregation. The 6:2 asci arise from crossing-over in either of the interstitial regions between centromeres and breakpoints, followed by alternate or adjacent 1 segregation (Perkins 1997). The eight-spored asci from *T(IBj5)^Nt^ A* × 85 *a* and *T* (*B362i*)*^Nt^ A* × 85 *a* included 8:0, 6:2, and 4:4 types (Table 4), and showed that both alternate and adjacent 1 segregation occur at comparable frequencies. Therefore, the absence of 8:0 and 6:2 asci from *T(IBj5)^Nt^ a* × *E A* and *T(B362i)^Nt^ A* × *E a* was quite surprising (Table 4). In these crosses, the viable ascospores came only from the 4:4 and 2:6 asci. Because the genome of both the *C4,T4 a*, and *E* strains is partly derived from the *N. tetrasperma* 343.6 *A* strain (Metzenberg and Ahlgren 1969), we suggest that the *T(IBj5)^Nt^ a* × *E A* and *T(B362i)^Nt^ A* × *E a* crosses may have become homozygous for a 343.6 *A*–derived recessive mutation that specifically affects alternate segregation, whereas the mutation may be heterozygous in the crosses *T* (*EB4*)*^Nt^ a* × *E A* and *T* (*UK14-1*)*^Nt^ A* × *E a*. To our best knowledge, such a mutant phenotype has not previously been reported. It is not detectable in an *N* × *N* cross, and an *RT* × *N* cross in the mutant background would produce only inviable ascospores. Given the possibility that *T(IBj5)* might be a complex rearrangement that includes a (I ; IV) *RT* (see *Materials and Methods*), homozygosity for the mutation can explain why *T*(*IBj5*)*^Nt^a* × *T*(*B362i*)*^Nt^A* yielded only inviable ascospores (Table 5). In principle, it should be possible by genome sequencing to identify a *343.6 A*–derived mutation present in *T(IBj5)^Nt^ a*, *T(B362i)^Nt^ A* and *E*, but absent in *T* (*EB4*)*^Nt^a* or *T* (*UK14-1*)*^Nt^A*.

The *Dp(EB4)*-borne *ad-7* gene was alterable by RIP in a *Dp(EB4)* × *E* cross. Our results suggest that when a duplicated segment undergoes a single round of RIP, one copy of it suffers G to A mutations, whereas the other suffers C to T mutations. Because self-crosses of [*Dp* + *Df*], but not [*T* + *N*], heterokaryons can undergo RIP, the extent to which a translocated segment is altered would depend on the number of adjacent 1 *vs.* alternate segregations in “ancestral self-crosses.”

All four [*T* + *N*] heterokaryons (*i.e.*, *EB4*, *IBj5*, *UK14-1*, and *B362i*) and three of the [*Dp* + *Df*] heterokaryons (*i.e.*, *EB4*, *IBj5*, and *UK14-1*) produced both self-fertile and self-sterile conidial derivatives; in most cases, the proportion of self-fertile conidial derivatives was >50% (Table 2). The [*N a + T(B362i) A*] and [*N a + Dp(B362i) A*] heterokaryons also produced self-fertile and self-sterile conidial derivatives, although in the [*N a + T(B362i) A*] strains the proportion of self-fertile ;conidial derivatives was <50%. Therefore, the inability of all seven [*Dp(B362i) a* + *Df(B362i) A*] strains examined to produce any self-fertile conidial derivatives was exceptional. It may be that the *Df(B362i) A* nuclei divide much less efficiently than the *Dp(B362i) a* nuclei; therefore, their fraction rapidly dwindles in the [*Dp(B362i) a* + *Df(B362i) A*] hyphae, making them increasingly less likely to be packaged along with the *Dp(B362i) a* nuclei during conidiation. Even if a few heterokaryotic [*Dp(B362i) a* + *Df(B362i) A*] conidia did form, the *Df(B362i) A* nuclei would be less likely to divide on conidial germination, thus biasing even the heterokaryotic conidia to produce homokaryotic *Dp(B362i) a* germlings. This hypothesis provides an explanation for an otherwise perturbing result that we had obtained in the early stages of this study. A strain was initially scored as [*Dp(B362i)* + *Df(B362i)*], because of its self-fertility and the ability of its DNA to support PCR amplification of all three *B362i*-specific junction fragments. However, later it behaved like a *Dp(B362i)* homokaryon, because it had become self-sterile and its newly isolated DNA failed to amplify the A fragment (D. A. Giri, unpublished results). A self-cross selectively restores the *Df(B362i)* nuclear fraction back to 50%, but self-crosses are not possible once all the *Df(B362i)* nuclei are lost. One model to account for the putative nuclear division defect of *Df(B362i) A* nuclei in the [*Dp(B362i) a* + *Df(B362i) A*] hyphae is that introgression of *T(B362i)*-linked chromosome 1 sequences from *N. crassa* disrupts the internuclear interactions of the *N. tetrasperma mat A* and *mat a* nuclei that were presumed to have co-evolved to optimize their fitness in [*mat A* + *mat a*] heterokaryons (Samils *et al.* 2014). The mating-type chromosome of *N. tetrasperma* has a recombination (crossing over) block over most of its length that ensures that ascospores get one nucleus of each mating type (Gallegos *et al.* 2000). The block has been correlated with large inversions on the *mat A* chromosome relative to the *mat a* chromosome, which has a gene arrangement like that of *N. crassa* (Ellison *et al.* 2011). The introgression of *T(B362i) A* chromosome 1 sequences might produce a *N. tetrasperma mat A* chromosome with more synteny with the *mat a* chromosome over most or all of its length, thereby disrupting the interactions between the *mat A* and *mat a* nuclei. Another model is that the *Df(B362i)* nuclei might simply lack a gene required for efficient nuclear division and whose null phenotype is not complemented by the wild-type allele in the neighboring *Dp(B362i)* nuclei, that is, a nucleus-limited gene. Studies showing the nucleus-limited nature of DNA damage checkpoint signal in *S. cerevisiae* (Demeter *et al.* 2000) are germane to the second model because they demonstrated that of two nuclei sharing the same cytoplasm, the one with damaged DNA arrests in mitosis without impeding progression through mitosis of the other with undamaged DNA. If the “disruption of co-evolved interactions” model is correct, then we might expect to see the same or similar phenotype following the introgression of another *N. crassa IT* whose recipient chromosome is chromosome 1, but if the “deletion of nucleus-limited gene” model is correct, then this phenotype might be *T(B362i)*-specific.

Introgression of additional *N. crassa* translocations into *N. tetrasperma* holds out the prospect of uncovering more genotypes with nucleus-limited effects. Alternatively, *N. tetrasperma* can now be transformed (Kasbekar 2015); therefore, a quicker way to screen for nucleus-limited genes might be to engineer targeted integration of yeast *Frt* sites into the different chromosome arms, and then use FLP recombinase to induce crossover and produce defined *RT*s. The [*Dp1*/*Df2* + *Dp2*/*Df1*] and [*RT* + *N*] heterokaryons emerging from *RT* × *N* crosses would enable us to screen two (or more) *Df*s in a single experiment.

## Supplementary Material

Supporting Information

## References

[bib1] Burton E. G. Metzenberg R. L., 1972 Novel mutation causing derepression of several enzymes of sulfur metabolism in *Neurospora crassa*. J. Bacteriol. 109: 140–151. 4257980 10.1128/jb.109.1.140-151.1972 PMC247261

[bib2] Calhoun F. Howe H. B. Jr, 1968 Genetic analysis of eight-spored asci produced by gene *E* in *Neurospora tetrasperma*. Genetics 60: 449–459. 5728739 10.1093/genetics/60.3.449 PMC1212054

[bib3] Corcoran P. Jacobson D. J. Bidartondo M. I. Hickey P. C. Kerekes J. F., 2012 Quantifying functional heterothallism in the pseudohomothallic ascomycete *Neurospora tetrasperma*. Fungal Biol. 116: 962–975. 22954339 10.1016/j.funbio.2012.06.006

[bib4] Czaja W. Miller K. Y. Miller B. L., 2013 Novel sexual-cycle specific gene silencing in *Aspergillus nidulans*. Genetics 193: 1149–1162. 23341415 10.1534/genetics.112.147546 PMC3606093

[bib5] Davis R. H., 1960 Adaptation in pantothenate-requiring Neurospora. II. Nuclear competition during adaptation. Am. J. Bot. 47: 648–654.

[bib6] Davis R. H. De Serres F. J., 1970 Genetic and microbiological research techniques for *Neurospora crassa*. Methods Enzymol. 17: 79–143.

[bib7] Demeter J. Lee S. E. Haber J. E. Stearns T., 2000 The DNA damage checkpoint signal in budding yeast is nuclear limited. Mol. Cell 6: 487–492. 10983994 10.1016/s1097-2765(00)00047-2

[bib8] Ellison C. E. Stajich J. E. Jacobson D. J. Nativig D. O. Lapidus A., 2011 Massive changes in genome architecture accompany the transition to self-fertility in the filamentous fungus *Neurospora tetrasperma*. Genetics 189: 55–69. 21750257 10.1534/genetics.111.130690 PMC3176108

[bib9] Gallegos A. Jacobson D. J. Raju N. B. Skupski M. P. Natvig D. O., 2000 Suppressed recombination and a pairing anomaly on the mating-type chromosome of *Neurospora tetrasperma*. Genetics 154: 623–633. 10655216 10.1093/genetics/154.2.623 PMC1460935

[bib10] Iyer S. V. Ramakrishnan M. Kasbekar D. P., 2009 *Neurospora crassa fmf-1* encodes the homologue of the *Schizosaccharomyces pombe* Ste11p regulator of sexual development. J. Genet. 88: 33–39. 19417542 10.1007/s12041-009-0005-2

[bib11] Jinks J. L., 1952 Heterokaryosis: a system of adaptation in wild fungi. Proc. R. Soc. Lond. B Biol. Sci. 140: 83–99. 13003914 10.1098/rspb.1952.0046

[bib12] Kasbekar D. P., 2013 Neurospora duplications, and genome defense by RIP and meiotic silencing, pp. 109–127 in Neurospora: Genomics and Molecular Biology, edited by Kasbekar D. P. McCluskey K. Caister Academic Press, Norfolk, UK.

[bib13] Kasbekar D. P., 2014 Are any fungal genes nucleus-limited? J. Biosci. 39: 341–346. 24845498 10.1007/s12038-014-9419-y

[bib14] Kasbekar D. P., 2015 What have we learned by doing transformations in *Neurospora tetrasperma*? pp. 47–52 in Genetic Transformation Systems in Fungi, Vol. 2, edited by van den Berg M. A. Maruthachalam K. Springer International Publishing, Switzerland.

[bib37] Kasbekar D. P. Singh P. K. Ramakrishnan M. Raj K. B., 2011 Carrefour Mme. Gras: A wild-isolated *Neurospora crassa* strain that suppresses meiotic silencing by unpaired DNA and uncovers a novel ascospore stability defect. Fungal Genet. Biol. 48: 612–620. 21295150 10.1016/j.fgb.2011.01.012

[bib15] Metzenberg R. L., 2003 Vogel’s Medium N salts: avoiding the need for ammonium nitrate. Fungal Genet. Newsl. 50: 14.

[bib16] Metzenberg R. L. Ahlgren S. K., 1969 Hybrid strains useful in transferring genes from one species of Neurospora to another. Neurospora Newslett. 15: 9–10.

[bib17] Newmeyer D., 1970 A suppressor of the heterokaryon-incompatibility associated with mating type in *Neurospora crassa*. Can. J. Genet. Cytol. 12: 914–926. 5512565 10.1139/g70-115

[bib18] Perkins D. D., 1991 Transfer of genes and translocations from *Neurospora crassa* to *N. tetrasperma*. Fungal Genet. Newsl. 38: 84.

[bib19] Perkins D. D., 1997 Chromosome rearrangements in Neurospora and other filamentous fungi. Adv. Genet. 36: 239–398. 9348657 10.1016/s0065-2660(08)60311-9

[bib20] Perkins D. D. Margolin B. S. Selker E. U. Haedo S. D., 1997 Occurrence of repeat induced point mutation in long segmental duplications of Neurospora. Genetics 147: 125–136. 9286673 10.1093/genetics/147.1.125 PMC1208096

[bib21] Perkins D. D. Radford A. Sachs M. S., 2001 The Neurospora Compendium Chromosomal Loci, Academic Press, San Diego.

[bib22] Raju N. B., 1979 Cytogenetic behavior of Spore killer genes in Neurospora. Genetics 93: 607–623. 17248974 10.1093/genetics/93.3.607 PMC1214101

[bib23] Raju N. B., 1980 Meiosis and ascospore genesis in Neurospora. Eur. J. Cell Biol. 23: 208–223. 6450683

[bib24] Raju N. B., 1986 Ascus-development in two temperature-sensitive Four-spore mutants of *Neurospora crassa*. Can. J. Genet. Cytol. 28: 982–990. 2950989 10.1139/g86-136

[bib25] Raju N. B., 1992 Functional heterothallism resulting from homokaryotic conidia and ascospores in *Neurospora tetrasperma*. Mycol. Res. 96: 103–116.

[bib26] Raju N. B. Perkins D. D., 1994 Diverse programs of ascus development in pseudohomothallic strains of Neurospora, Gelasinospora, and Podospora. Dev. Genet. 15: 104–118. 8187347 10.1002/dvg.1020150111

[bib27] Raju N. B. Metzenberg R. L. Shiu P. K. T., 2007 Neurospora spore killers Sk-2 and Sk-3 suppress meiotic silencing by unpaired DNA. Genetics 176: 43–52. 17339226 10.1534/genetics.106.069161 PMC1893035

[bib28] Ramakrishnan M. Sowjanya T. N. Raj K. B. Kasbekar D. P., 2011 Meiotic silencing by unpaired DNA is expressed more strongly in the early than the late perithecia of crosses involving most wild-isolated *Neurospora crassa* strains and in self-crosses of *N. tetrasperma*. Fungal Genet. Biol. 48: 1146–1152. 22056520 10.1016/j.fgb.2011.10.002

[bib29] Rieger R. Michaelis A. Green M. M., 1991 Glossary of Genetics Classical and Molecular, Ed. 5th Springer-Verlag, Berlin.

[bib30] Samils N. Oliva J. Johannesson H., 2014 Nuclear interactions in a heterokaryon: insight from the model *Neurospora tetrasperma*. Proc. Biol. Sci. 281: 20140084. 24850920 10.1098/rspb.2014.0084 PMC4046401

[bib31] Shiu P. K. Glass N. L., 1999 Molecular characterization of *tol*, a mediator of mating-type-associated vegetative incompatibility in *Neurospora crassa*. Genetics 151: 545–555. 9927450 10.1093/genetics/151.2.545 PMC1460514

[bib32] Shiu P. K. Raju N. B. Zickler D. Metzenberg R. L., 2001 Meiotic silencing by unpaired DNA. Cell 107: 905–916. 11779466 10.1016/s0092-8674(01)00609-2

[bib33] Selker E. U., 1990 Premeiotic instability of repeated sequences in *Neurospora crassa*. Annu. Rev. Genet. 24: 579–613. 2150906 10.1146/annurev.ge.24.120190.003051

[bib34] Singh P. K. Iyer S. V. Sowjanya T. N. Raj B. K. Kasbekar D. P., 2010 Translocations used to generate chromosome segment duplications in Neurospora can disrupt genes and create novel open reading frames. J. Biosci. 35: 539–546. 21289436 10.1007/s12038-010-0062-y

[bib35] Singh, P. K., 2010 Genetic and molecular analysis of Neurospora duplications and duplication-generating translocations. PhD Thesis, Jawaharlal Nehru University, New Delhi.

[bib36] Smith M. L. Lafontaine D. L., 2013 The fungal sense of non-self, pp. 9–21 in Neurospora: Genomics and Molecular Biology, edited by Kasbekar D. P. McCluskey K. Caister Academic Press, Norfolk, UK.

