# Supplementary material for: Neurospora Heterokaryons with Complementary Duplications and Deficiencies in Their Constituent Nuclei Provide an Approach to Identify Nucleus-Limited Genes
Source: G3 (Bethesda). 2015 Apr 20;5(6):1263–72. doi: 10.1534/g3.115.017616 (PMC4478554; doi:10.1534/g3.115.017616)
Supplement: Supporting Information [file supp_5_6_1263__index.html]

Neurospora Heterokaryons with Complementary Duplications and Deficiencies in Their Constituent Nuclei Provide an Approach to Identify Nucleus-Limited Genes — Supporting Information 

# *Neurospora* Heterokaryons with Complementary Duplications and Deficiencies in Their Constituent Nuclei Provide an Approach to Identify Nucleus-Limited Genes

## Supporting Information for Giri, Rekha, and Kasbekar, 2015

**Files in this Data Supplement:**

- Supporting Information - Figure S1 and Table S1 (PDF, 1 MB)
- Figure S1 - *C4,T4 a* is a weak MSUD suppressor. (PDF, 1010 KB)
- Table S1 - Oligonucleotide primers used for PCR. (PDF, 40 KB)
