# Supplementary material for: Neurospora Heterokaryons with Complementary Duplications and Deficiencies in Their Constituent Nuclei Provide an Approach to Identify Nucleus-Limited Genes
Source: G3 (Bethesda). 2015 Apr 20;5(6):1263–72. doi: 10.1534/g3.115.017616 (PMC4478554; doi:10.1534/g3.115.017616)
Supplement: Supporting Information [file supp_g3.115.017616_017616SI.pdf]

G3 · Genes | Genomes | Genetics

Title: Neurospora heterokaryons with complementary duplications and deficiencies in their constituent nuclei provide an approach to identify nucleus-limited genes.

Authors: Dev Ashish Giri[1, 2], S. Rekha[1] and Durgadas P. Kasbekar[1]

Institutional Affiliation: [1]Centre for DNA Fingerprinting and Diagnostics, Hyderabad 500001, India, [2]Graduate Studies, Manipal University, India.

Accession numbers of nucleotide sequences determined in this work: KP006652; KP006653; KP006654

Corresponding author: Durgadas P. Kasbekar, Centre for DNA Fingerprinting and Diagnostics, Tuljaguda Complex, Nampally, Hyderabad 500001, India. Phone +91-40-24749401; Email kas@cdfd.org.in

DOI: 10.1534/g3.115.017616

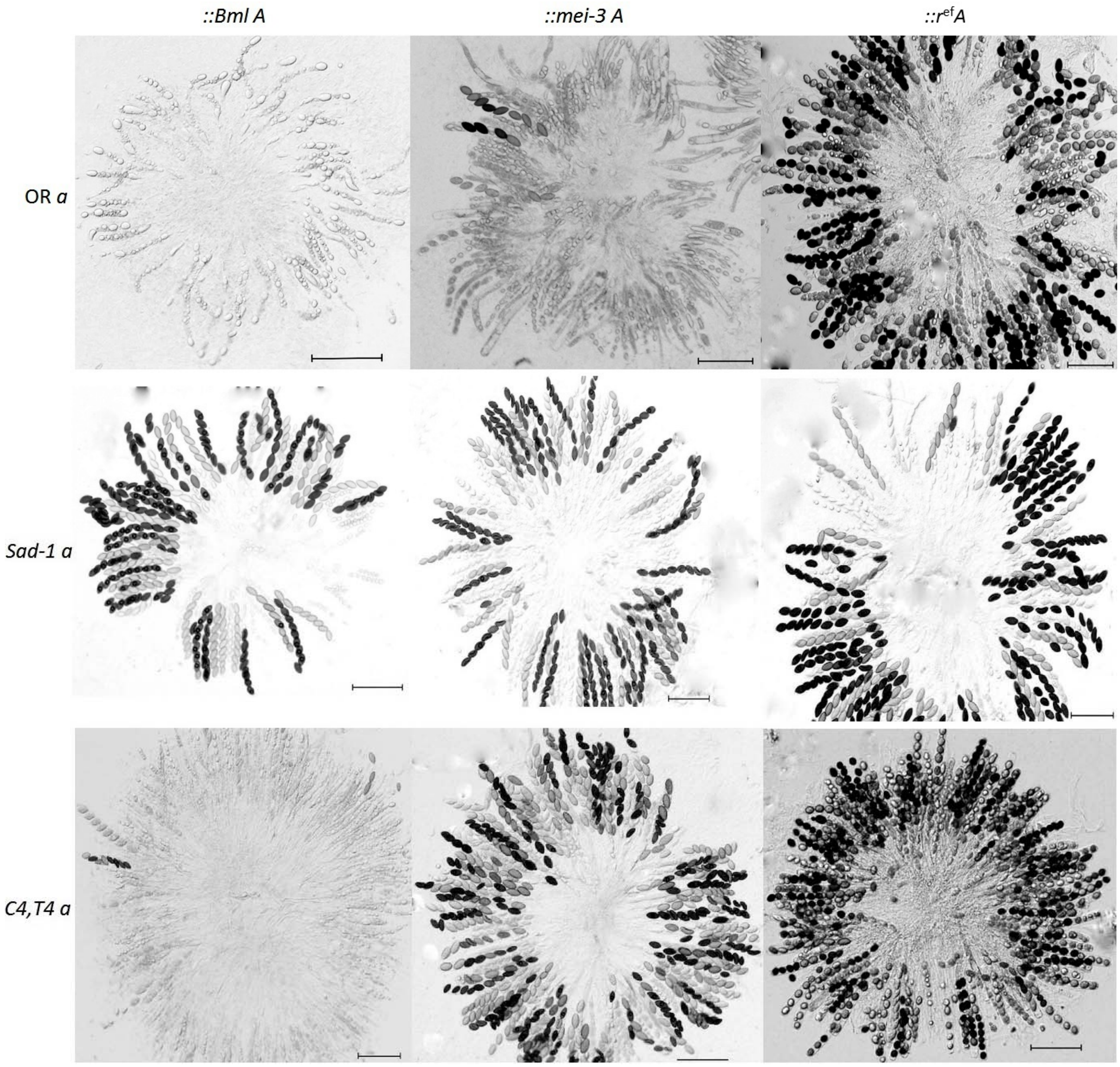

Figure S1 *C4,T4 a* is a weak MSUD suppressor. Ascus development in crosses of the *N. crassa* strains OR *a* and *Sad-1 a*, and the *N. crassa* / *N. tetrasperma* hybrid strain *C4,T4 a* with the MSUD tester strains *::bml A*, *::mei-3 A* and *::r^{ef2}*. Meiotic silencing of the *bml* (β-tubulin) and *mei-3* genes in the crosses with OR *a* disrupts ascus development, whereas its suppression in the crosses with *Sad-1 a* allows normal ascus development. Silencing of *r* in the cross with OR *a* causes all eight ascospores to be round, and its suppression by *Sad-1 a* restores the normal spindle shape. Silencing is evident in crosses of *C4,T4 a* with *::bml A* and *::r^{ef2}A* but not in the cross with *::mei-3 A*. Partial suppression of MSUD by *C4,T4 a* is characteristic of Esm type strains (Ramakrishnan *et al*., 2011).

Table S1 Oligonucleotide primers used for PCR.

Translocation	Breakpoint		
	A	B	C
**EB4**	GCCGGTTTTGGAGCA TCCATACACAGGG	GCGGGCGGCAAA GGCTGTT	TCCACACCAGAG GTCGTAG
	AAGTTGTAGCTACGC TGAAACACCAGATG ACC	GGGCGGCTTCGGC AGTAAA	ATCAATTCGCGA TGGAACGG
**IBj5**	CTCTCGCCCGACTAG GACTTC	GCTGTGACTCATA CTTCCCCC	-
	GTTGCCCTGCTTTCC GTGCG	GTTCGCTAGTGAG TGCGTTCC	
**UK14-1**	GGTAGGTAAGGAAG GTGCAATCG	-	NA
	CGATGAAGAGAGGC CCAGTGAAGAC		
**B362i**	ATAGTGGGAGCTGTC ACAGGTTCCCTTG	AGCTCGAATCGCG AGGAGAG	TTCATCGAGACC GGCTGGAAG
	AAGTTGTAGCTACGC TGAAACACCAGATG ACC	GTCTTCGGGCTTC AACCGAG	CGCGATGTCACC GACGAAAG

NA – not applicable.

Oligonucleotide primers used to PCR amplify *ad-7* sequences of *RIP3C* and *RIP3T*

RIP3C	CTCCAACCTAGCACTTGTTCGATC
	GGTTGAGGCTGATATCCATTCTCC
RIP3T	GTATGTATCCGTCCCCTCTCATCTC
	GAAGCCAAAGCAATCGGTCG
