# Supplementary material for: Neurospora Heterokaryons with Complementary Duplications and Deficiencies in Their Constituent Nuclei Provide an Approach to Identify Nucleus-Limited Genes
Source: G3 (Bethesda). 2015 Apr 20;5(6):1263–72. doi: 10.1534/g3.115.017616 (PMC4478554; doi:10.1534/g3.115.017616)
Supplement: Supporting Information [file supp_g3.115.017616_FigureS1.pdf]

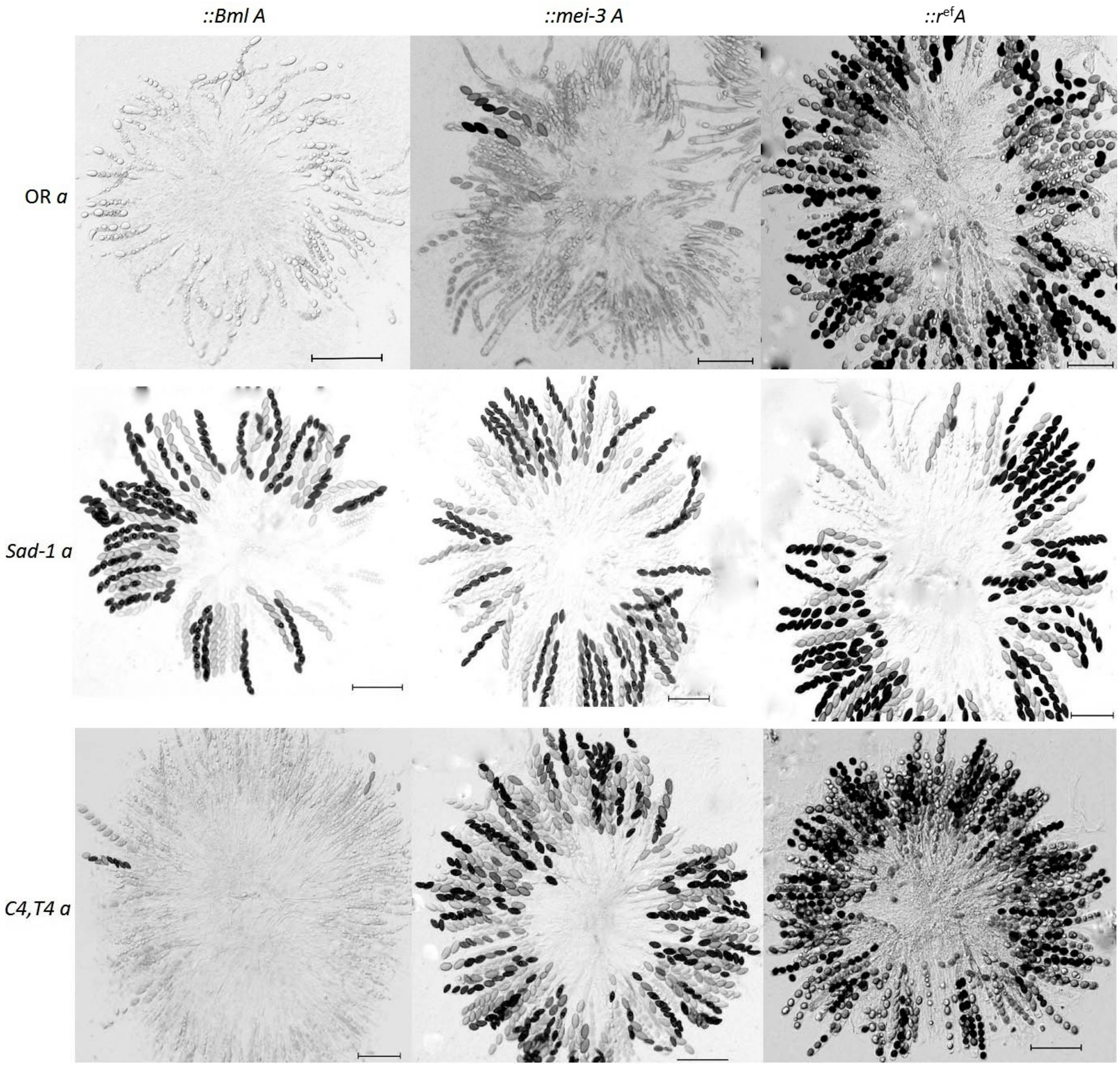

Figure S1 *C4,T4 a* is a weak MSUD suppressor. Ascus development in crosses of the *N. crassa* strains OR *a* and *Sad-1 a*, and the *N. crassa* / *N. tetrasperma* hybrid strain *C4,T4 a* with the MSUD tester strains *::bml A*, *::mei-3 A* and *::r^{ef2}*. Meiotic silencing of the *bml* (β-tubulin) and *mei-3* genes in the crosses with OR *a* disrupts ascus development, whereas its suppression in the crosses with *Sad-1 a* allows normal ascus development. Silencing of *r* in the cross with OR *a* causes all eight ascospores to be round, and its suppression by *Sad-1 a* restores the normal spindle shape. Silencing is evident in crosses of *C4,T4 a* with *::bml A* and *::r^{ef2}A* but not in the cross with *::mei-3 A*. Partial suppression of MSUD by *C4,T4 a* is characteristic of Esm type strains (Ramakrishnan *et al*., 2011).
