# Supplementary material for: Neurospora Heterokaryons with Complementary Duplications and Deficiencies in Their Constituent Nuclei Provide an Approach to Identify Nucleus-Limited Genes
Source: G3 (Bethesda). 2015 Apr 20;5(6):1263–72. doi: 10.1534/g3.115.017616 (PMC4478554; doi:10.1534/g3.115.017616)
Supplement: Supporting Information [file supp_g3.115.017616_TableS1.pdf]

Table S1 Oligonucleotide primers used for PCR.

Translocation	Breakpoint		
	A	B	C
**EB4**	GCCGGTTTTGGAGCATCCATACACAGGG	GCGGGCGGCAAAGGCTGTT	TCCACACCAGAGGTCGTAG
	AAGTTGTAGCTACGCTGAAACACCAGATGACC	GGGCGGCTTCGGCAGTAAA	ATCAATTCGCGATGGAACGG
**IBj5**	CTCTCGCCCGACTAGGACTTC	GCTGTGACTCATACTTCCCCC	-
	GTTGCCCTGCTTTCCGTGCG	GTTCGCTAGTGAGTGCGTTCC	
**UK14-1**	GGTAGGTAAGGAAGGTGCAATCG	-	NA
	CGATGAAGAGAGGCCCAGTGAAGAC		
**B362i**	ATAGTGGGAGCTGTCACAGGTTCCCTTG	AGCTCGAATCGCGAGGAGAG	TTCATCGAGACCGGCTGGAAG
	AAGTTGTAGCTACGCTGAAACACCAGATGACC	GTCTTCGGGCTTCAACCGAG	CGCGATGTCACCGACGAAAG

NA – not applicable.

Oligonucleotide primers used to PCR amplify *ad-7* sequences of *RIP3C* and *RIP3T*

RIP3C	CTCCAACCTAGCACT<br>TGTTCGATC
	GGTTGAGGCTGATAT<br>CCATTCTCC
RIP3T	GTATGTATCCGTCCC<br>CTCTCATCTC
	GAAGCCAAAGCAAT<br>CGGTCG
